# A Clinical Conversation

**Published:** 1889-08

**Authors:** E. W. Berridge

**Affiliations:** London


### A CLINICAL CONVERSATION.

E. W. Berridge, M. D., London.

A few days ago I was conversing with my friend and preceptor, Dr. David Wilson, and his remarks were so interesting that I wrote them down at the time, and read them to him to insure accuracy.

He first called my attention to symptoms 731 and 744 of *Sulphuric acid*: "Respiration rapid, with shooting in cervical muscles, and *movement of the wings of nose*." "Respiration became very difficult; *the larynx moved up and down violently;* the child lay with the head bent backward, as in the last stages of croup; he lost consciousness, and soon died." The fanlike action of the nostrils was pointed out by Dr. Wilson as characteristic of *Lycopodium* about 1862; and he subsequently told me that the up and down movement of the larynx belonged clinically to the same medicine. Under *Sulphuric acid* we find both these symptoms; but, as they occurred in cases of poisoning by the strong acid, it is necessary to ascertain whether these were the result of the dynamic action of the drug, or the result of the shock to the system caused by its corrosive chemical action. This can be determined not only by further provings, but also by the clinical test. Dr. Wilson informed me that in a fatal case of Cheyne-Stokes respiration, *Sulphuric acid*$^{200}$ had removed this movement of the larynx, and somewhat ameliorated the abnormal respirations.

With regard to the symptom "stoppage of respiration," Dr. Wilson has verified, clinically, *Bryonia*, *Opium*, and *Sulphur*. He told me, moreover, that he had seen but five cases of Cheyne-Stokes respiration, and only one recovered, this patient had permanent mitral regurgitation; the olfaction of *Opium*$^{200}$ for five days, whenever the breathing stopped, saved him.

Several remedies have stoppage of respiration, but I cannot find any that have the exact symptom of this rare disease, viz.: respiration gradually increasing in strength, then gradually decreasing, with an interval of complete cessation before it begins again."

In Hempel's *Jahr* the following symptoms of *Croton* are given: "Violent ophthalmia; on second day, ulceration of the conjunctiva over the cornea (in two places), ulceration of the conjunctiva over sclerotica (in various parts), irritation of sclerotica and iris; contraction of pupil; injected state of vessels of conjunctiva sclerotica and eyelids, profuse lachrymation, photophobia, violent pains disturbing the night's rest; increased dimness of cornea on third day, increased depth of the ulcerated parts, rudimentary hypopyon in anterior chamber; on seventh day nothing remained of the inflammation except a slight irritation of the eye, and a slight dimness of the corneal portion of the conjunctiva in those places which had been ulcerated." This symptom, the source of which is not given, is omitted in Allen's *Encyclopædia*. Hering's *Guiding Symptoms* records it, omitting the hypopyon, and adding "burning of eye." Clearly, therefore, there is some error, and the original version should be sought for and properly translated.

But Dr. Wilson has verified this symptom in puppies eight weeks old, suffering from purulent ophthalmia, with great agglutination of lids, well marked hypopyon, and small indentations, as if cornea were commencing to ulcerate. One dose of *Croton*$^{30}$ cured.

Under *Æthusa*, Hempel's *Jahr* gives (under "Pathological Anatomy") "Bloated countenance; the cornea is dim and deeply sunken, the pupils are very much dilated." This symptom I cannot find in the *Encyclopædia*, but I saw Dr. Wilson cure the symptom, "sunken cornea," with one dose of *Æthusa*$^{1m}$ (Jenicken). This occurred at his dispensary, more than twenty years ago.

I will conclude these notes with a recent case of my own: On February 20th, 1889, a patient told me that her dog had purulent ophthalmia. He had been ill for a week, both eyes closed by yellow discharge; does not like to open them even when bathed; eyes red; lies close to the fire. The selection of the remedy was difficult, as I did not see the dog; but, remembering the intense photophobia of *Conium*, and that the dog was old, I selected this remedy; and, not being sure it was more than a *simile* (not, perhaps, a *simillimum*), I prescribed a dose of Cm (F. C.) in water, three times daily for eight days.

March 7th.—My patient reported that the dog improved slightly within a week; opened eyes seventh or eighth day. She says the left eye looks opaque, with a hole in it; right eye also partly opaque. I advised her to bring the dog to be inspected. This she did not do; but on April 12th she reported that the eyes had quite healed, and the hole had disappeared. Also that he seemed much better in himself generally, since taking the medicine. It was evidently a case of ulceration of cornea.

The homœopathic treatment of animals is of just interest, because

(1.) Our opponents cannot allege that they were cured by "faith" or "imagination," or even by "Christian Science."

(2.) They do not read works on Domestic Homœopathy, and then spoil the treatment by taking *Aconite* and *Belladonna* in alternation for some temporary ailment.

(3.) They are grateful for being cured, which is more than can be said of some patients, and—some colleagues.

Repertory, p. 29, "Experienced" for k-bi. and k-bro. Allen's Repertory has the same error.

